# Cementless versus cemented total knee arthroplasty in young patients: a meta-analysis of randomized controlled trials

**DOI:** 10.1186/s13018-019-1293-8

**Published:** 2019-08-19

**Authors:** Chengyu Chen, Ruodong Li

**Affiliations:** 1 Department of Orthopedics, People’s Hospital of Yuncheng, Heze, 274700 Shandong Province China; 2 Department of Orthopedics, People’s Hospital of Liaocheng Dongchangfu, Liaocheng, 252000 Shandong Province China

**Keywords:** Total knee arthroplasty, Cementless, Cemented, Meta-analysis

## Abstract

**Purpose:**

Optimal type of prosthesis in total knee arthroplasty (TKA) remains controversial for young patients. The objective of this meta-analysis is to compare cementless and cemented fixation in TKA.

**Methods:**

In this meta-analysis, we conducted electronic searches of PubMed, Embase, Cochrane Library, and Web of Science in December 2018. We collected randomized controlled trials (RCTs) comparing cementless and cemented TKA in young patients. The outcome measurements consisted of functional outcomes, Knee Society Score, range of motion, radiological outcomes, pain score, and complications. Stata 12.0 software was used for our meta-analysis. Quality assessment for RCTs was conducted according to the Cochrane Handbook for systematic review of interventions.

**Results:**

Four RCTs met our inclusion criteria with 255 patients in cemented groups and 229 patients in cementless groups. The present meta-analysis indicated that there was a significant difference between the groups in terms of radiological outcomes and pain score. No significant difference was found regarding KSS, range of motion, or complications.

**Conclusion:**

Cementless TKA was associated with superior outcomes in terms of radiological outcomes and pain score compared with cemented fixation. We found no significant difference regarding the functional outcome or aseptic loosening between groups. High-quality RCTs were still required for further investigation.

## Introduction

Total knee arthroplasty (TKA) is a popular surgical procedure for treating knee osteoarthritis (OA) and rheumatoid arthritis (RA) [1, 2]. In the Australian Joint Replacement Register, patient demographics in TKA are slowly changing to younger patients, with the number of patients < 65 years of age increased by 40% from the start of the register in 2002 until 2007 [3]. The demand and stress are higher for young patients because of the longer life expectancy and higher quality of daily life [4]. For younger patients, the failure of TKA is commonly due to the lack of fixation of tibial side implant and periprosthetic infection. Aseptic loosening is a major complication after TKA which may cause high revision risk [5]. Currently, the optimal type of prosthesis remains controversial for young patients.

Cement prosthesis enhances early fixation and prevents the periprosthetic bone resorption [6]. Cemented TKA has been the gold standard in TKA with improved outcome and implant survivorship as long as 20 years [7]. However, toxic effect may be a major concern and it is more difficult for revision surgery. Besides, young patients have a higher demand of stresses on implants. Cementless fixation in TKA has become more and more popular because it is associated with a long term of survival, particularly in younger patients. Previous studies reported that use of cementless fixation could achieve a physiological bond between bone and implant which results in a prolonged survival from aseptic loosening [8, 9]. However, evidence of osteolysis has also been shown with cementless implants; thus, it has not been widely accepted in the field of joint surgery. Few randomized controlled trials (RCT) have compared the clinical outcomes of cemented versus cementless TKA. It is unknown for us whether cemented prosthesis is superior to cementless prosthesis. Therefore, we perform the meta-analysis from recent published RCTs to evaluate the optimal mode of fixation in young patients’ TKA.

## Methods

The work has been reported in line with PRISMA (Preferred Reporting Items for Systematic Reviews and Meta-Analyses).

### Literature search

The following electronic databases were independently and extensively searched by two investigators from their inception through December 2018: Embase, Medline, the Cochrane Library, and Web of Science. The search keywords were centered on the terms “total knee arthroplasty OR total knee replacement,” “cementless,” and “cemented,” which were adjusted to each database in necessity. In addition, the bibliographies of the included studies and dissertations were searched for additional publications. The search language was restricted to English.

### Inclusion and exclusion criteria

Eligible studies were considered if they met the following criteria: (1) population: patients aged 60 years or younger who received TKA; (2) intervention: cementless prosthesis; (3) comparison: cemented prosthesis; (4) outcome measures: at least one of the following outcome measures was reported: functional outcomes, radiological evaluation, pain score, and complication; and (5) study design: only RCTs. We excluded articles that were (1) duplicate reports and conference abstracts, (2) articles without available full-text versions, (3) no available outcomes data, and (4) review or case report.

### Data extraction

Two researchers independently extracted the data from the included literature. The corresponding author was consulted for details in the case of incomplete data. The following information was extracted: first author name, year of publication, intervening measures, comparable baseline, sample size, and outcome measures. Other relevant parameters were also extracted from individual studies.

### Risk of bias assessment

Quality assessment for RCT was conducted according to the Cochrane Handbook for systematic review of interventions. To provide a qualification of bias risk, quality criteria included (i) details of randomization method, (ii) allocation concealment, (iii) blinding of participants and personnel, (iv) blind outcome assessment, (v) incomplete outcome data, (vi) selective outcome reporting, and (vii) other sources of bias. Each aspect could further be classified as low, high, or unclear risk. The evidence grade was assessed using the guidelines of the Recommendations Assessment, Development and Evaluation (GRADE) system. Disagreements were resolved through a discussion with a third review author.

### Statistical analysis

Continuous outcomes were expressed as the weighted mean differences (WMD) with 95% CI. Statistical significance was set at *P* < 0.05 to summarize the findings across the trials. Variables in the meta-analysis were calculated using Stata software, version 12.0 (Stata Corp., College Station, TX). Statistical heterogeneity was evaluated using the chi-square test and the *I*^2^ statistic. When there was no statistical evidence of heterogeneity (*I*^2^ < 50%, *P* > 0.1), a fixed effects model was adopted; otherwise, a random effects model was chosen. Funnel plots to assess publication bias in the included studies were not constructed because of the limited number of studies.

## Result

### Search results

**Fig. 1:**
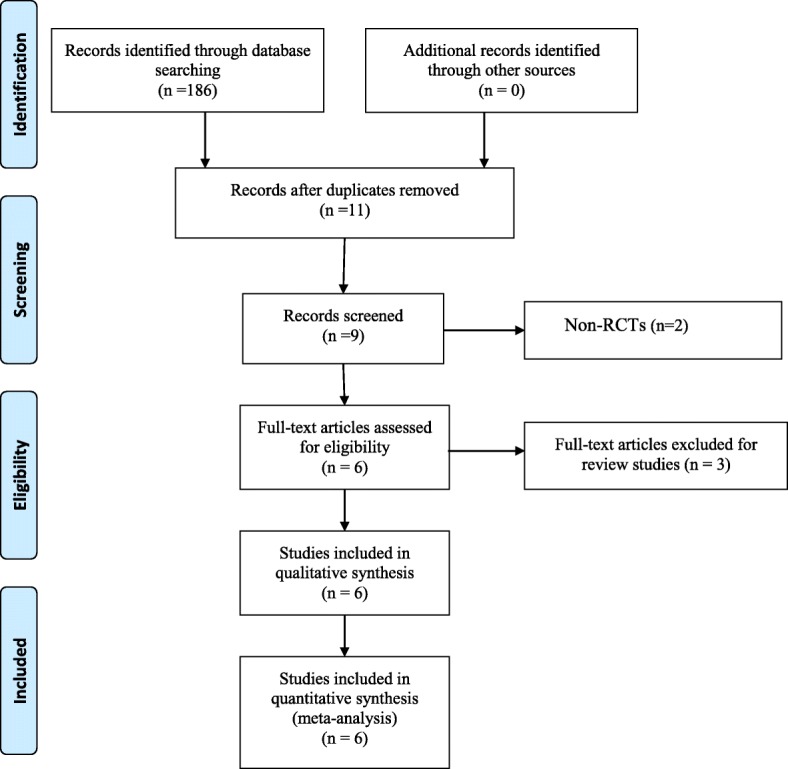
Flow chart of study selection

Figure 1 contains a flowchart that describes the process by which we screened and selected trials. The initial literature search yielded 186 articles in all. Duplicate checking and title and abstract screening resulted in 11 publications, and the full text of all 9 articles were available. Among these, 2 were excluded because the articles were non-RCTs. In addition, manual search of relevant reference did not identify any additional studies. Finally, 6 [10–15] studies were eligible for inclusion in this review.

### Study characteristics

The characteristic of the included RCTs is presented in Table 1. Of the included 484 patients, 255 patients were treated with cemented prosthesis and 229 patients were treated with cementless prosthesis. All RCTs were published between 2009 and 2016. The duration of follow-up ranged from 2 to 13.6 years. All RCTs did not use screws for additional reinforcement and resurfaced the patella.

**Table 1:** Characteristics of the included studies

Study	Year	Design	Study	Age		Gender (male %)		No. of patients		Outcomes	Mean follow-up
				Cemented	Cementless	Cemented	Cementless	Cemented	Cementless		
Nilsson et al. [10]	2006	RCT	TKA	56	56	42%	45%	34	35	KSS, radiological outcomes	24 months
Gao et al. [11]	2009	RCT	TKA	54	58	39%	44%	22	19	KSS, pain score, radiological outcomes, complication	24 months
Park et al. [12]	2011	RCT	TKA	59	61	36%	43%	50	50	KSS, range of motion, pain score, radiological outcomes, complication	13.6 years
Kim et al. [13]	2014	RCT	TKA	54	53	39%	41%	80	80	KSS, range of motion, radiological outcomes, complication	16.6 years
Lizaur-Utrilla et al. [14]	2012	RCT	TKA	52	51	47%	45%	48	45	KSS, range of motion, pain score, radiological outcomes, complication	7.2 years
Henricson et al. [15]	2013	RCT	TKA	56	54	46%	41%	21	26	KSS, range of motion, pain score, radiological outcomes, complication	10 years

*RCT* randomized controlled trial, *TKA* total knee arthroplasty

### Assessment of the methodological quality

**Fig. 2:**
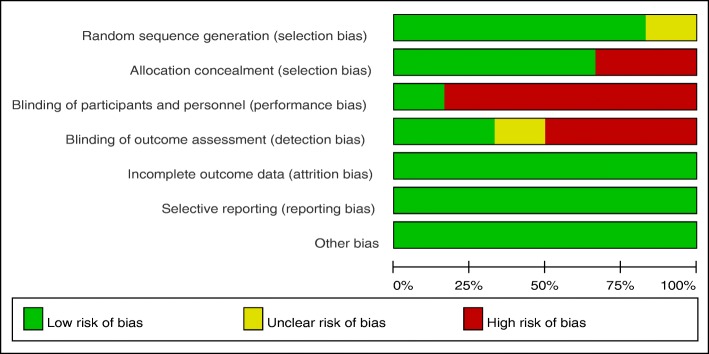
Risk of bias graph

The risk of bias assessed by the Cochrane tool in each included studies is shown in Table 2. All studies reported the randomization, and five of them adopted computer-generated random sequence. Four RCTs used a sealed opaque envelope for allocation concealment. Only one study explicitly described blinding of both patients and personnel, and two studies conducted blinding to the outcome assessors. All the studies clearly reported follow-up results to avoid reporting bias, although the follow-up intervals were not consistent. However, the shortcomings of these six studies were the lack of intention-to-treat analysis. Each risk of the bias item was expressed in terms of the percentage across all the included studies, which indicated the proportion of risk levels for each item bias (Fig. 2).

**Table 2:** Methodological quality of the randomized controlled trials

Study	Random sequence generation	Allocation concealment	Blinding of participants and personnel	Blinding of outcome assessment	Incomplete outcome data	Selective reporting	Other bias
Nilsson et al. [10]	Low risk	Low risk	High risk	Low risk	Low risk	Low risk	Low risk
Gao et al. [11]	Low risk	Low risk	High risk	Low risk	Low risk	Low risk	Low risk
Park et al. [12]	Low risk	Low risk	High risk	High risk	Low risk	Low risk	Low risk
Kim et al. [13]	Low risk	Low risk	High risk	High risk	Low risk	Low risk	Low risk
Lizaur-Utrilla et al. [14]	Low risk	High risk	Low risk	Low risk	Low risk	Low risk	Low risk
Henricson et al. [15]	Unclear risk	High risk	High risk	Unclear risk	Low risk	Low risk	Low risk

### Meta-analysis of functional recovery

**Fig. 3:**
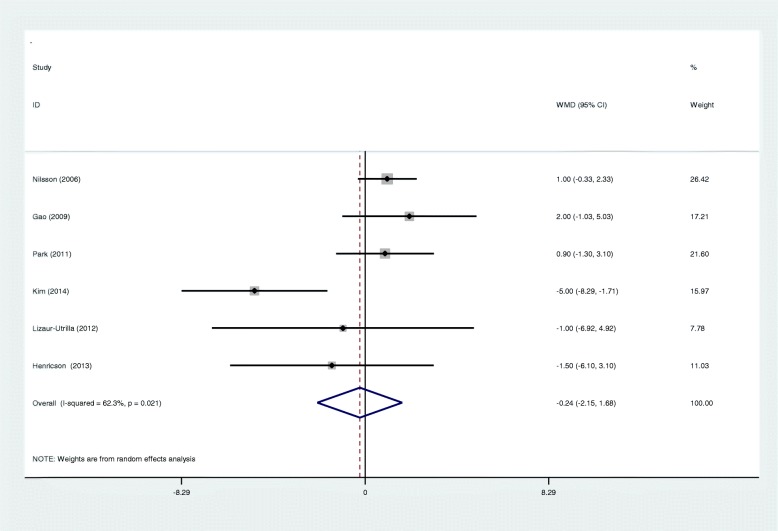
Forest plots for functional recovery

All included RCTs reported Knee Society Score (KSS). There was significant heterogeneity among studies (*I*^2^ = 62.3%, *p* = 0.021), and a random effects model was adopted. The pooled results showed there was no significant difference between the two groups in terms of functional outcome (WMD − 0.239; 95% CI − 2.154 to − 1.676; *p* = 0.807, Fig. 3).

### Meta-analysis of range of motion

**Fig. 4:**
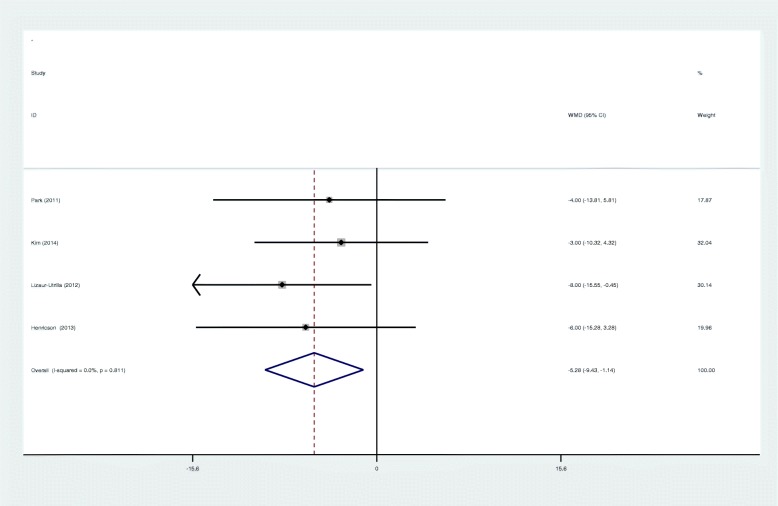
Forest plots for range of motion

Four studies provided a postoperative range of motion at 5 years’ follow-up. Meta-analysis showed the benefit of cementless fixation compared to cemented fixation in range of motion (ROM) (WMD − 5.284; 95% CI − 9.430 to − 1.139; *p* = 0.012, Fig. 4). A fixed effects model was adopted because no significant heterogeneity was found (*I*^2^ = 0%, *p* = 0.811).

### Meta-analysis of pain score

**Fig. 5:**
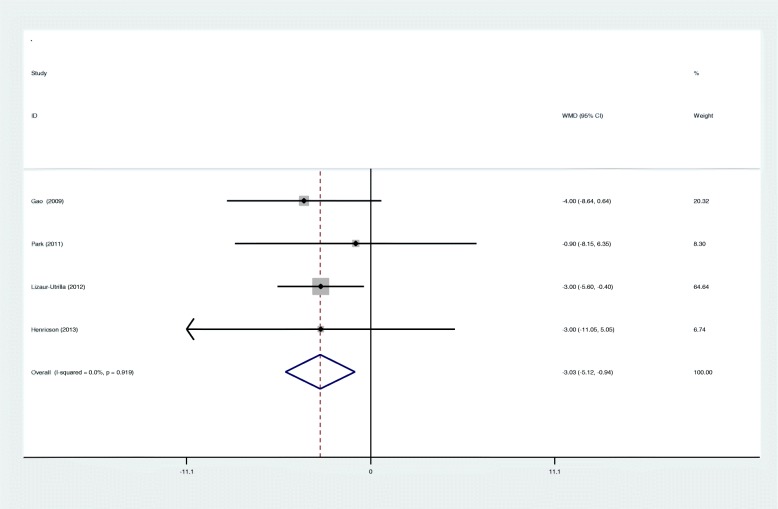
Forest plots for pain score

Four articles showed the outcome of postoperative pain score. There was no significant heterogeneity among studies (*I*^2^ = 0%, *p* = 0.919), and a fixed effects model was used. Our study showed that cementless fixation was associated with a significant improved pain score after TKA (WMD − 3.029; 95% CI − 5.119 to − 0.939; *p* = 0.005, Fig. 5).

### Meta-analysis of radiological outcomes

**Fig. 6:**
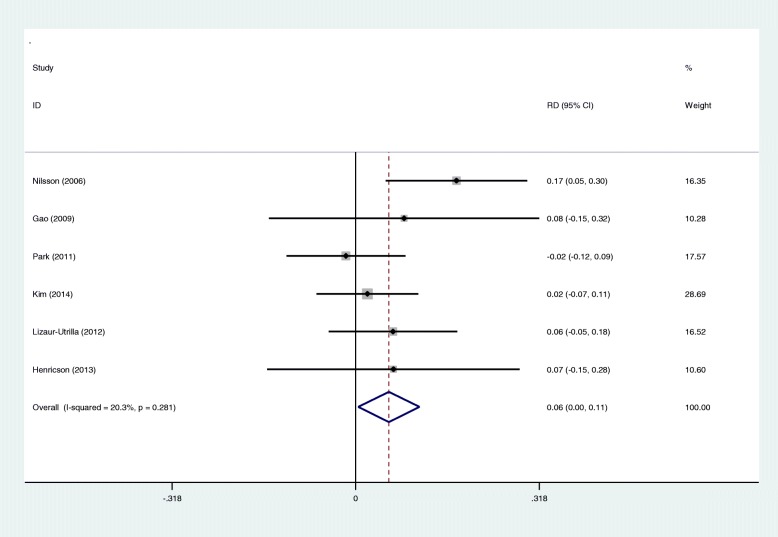
Forest plots for radiological outcomes

A total of six studies showed the radiolucent line (< 1 mm) in the tibial component side. There was no significant heterogeneity (*I*^2^ = 20.3%, *p* = 0.281), and a fixed effects model was used. There was significant difference between groups regarding the radiological outcomes (RD 0.058; 95% CI 0.004 to 0.111; *p* = 0.034, Fig. 6).

### Meta-analysis of aseptic loosening

**Fig. 7:**
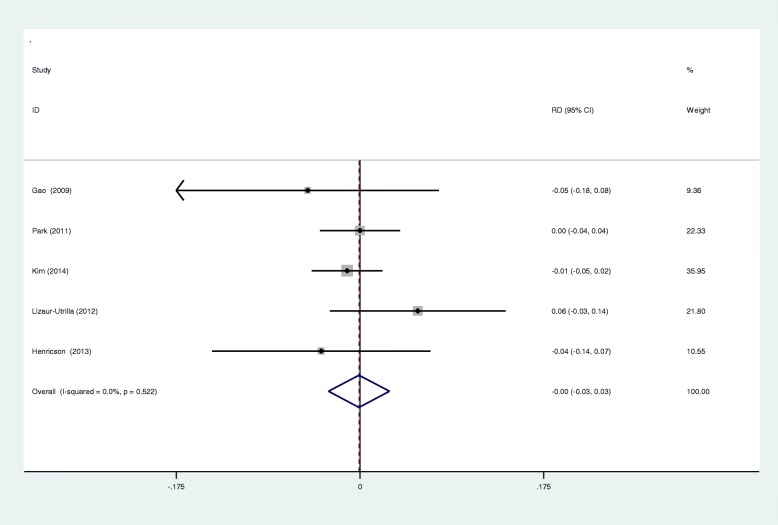
Forest plots for aseptic loosening

Aseptic loosening was reported in five RCTs. A fixed effects model was used (*I*^2^ = 0%, *p* = 0.522). We found that there was no significant difference between the groups regarding the incidence of aseptic loosening (RD − 0.001; 95% CI − 0.030 to 0.028; *p* = 0.946, Fig. 7).

### Meta-analysis of complications

**Fig. 8:**
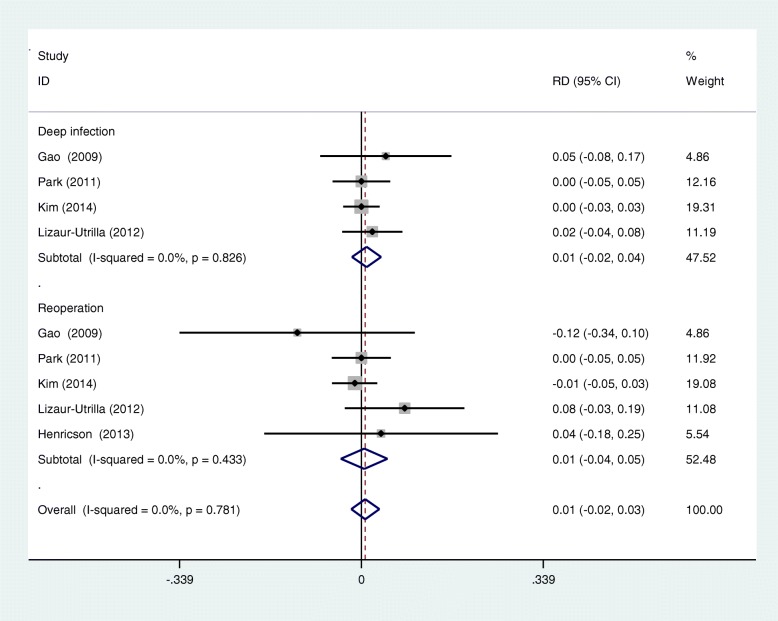
Forest plots for complications

Five RCTs reported the postoperative complication including deep infection and reoperation. A fixed effects model was used (*I*^2^ = 0%, *p* = 0.781). No significant difference was identified in terms of postoperative complication (RD 0.007; 95% CI − 0.019 to 0.033; *p* = 0.587, Fig. 8).

### Publication bias and evidence level

**Fig. 9:**
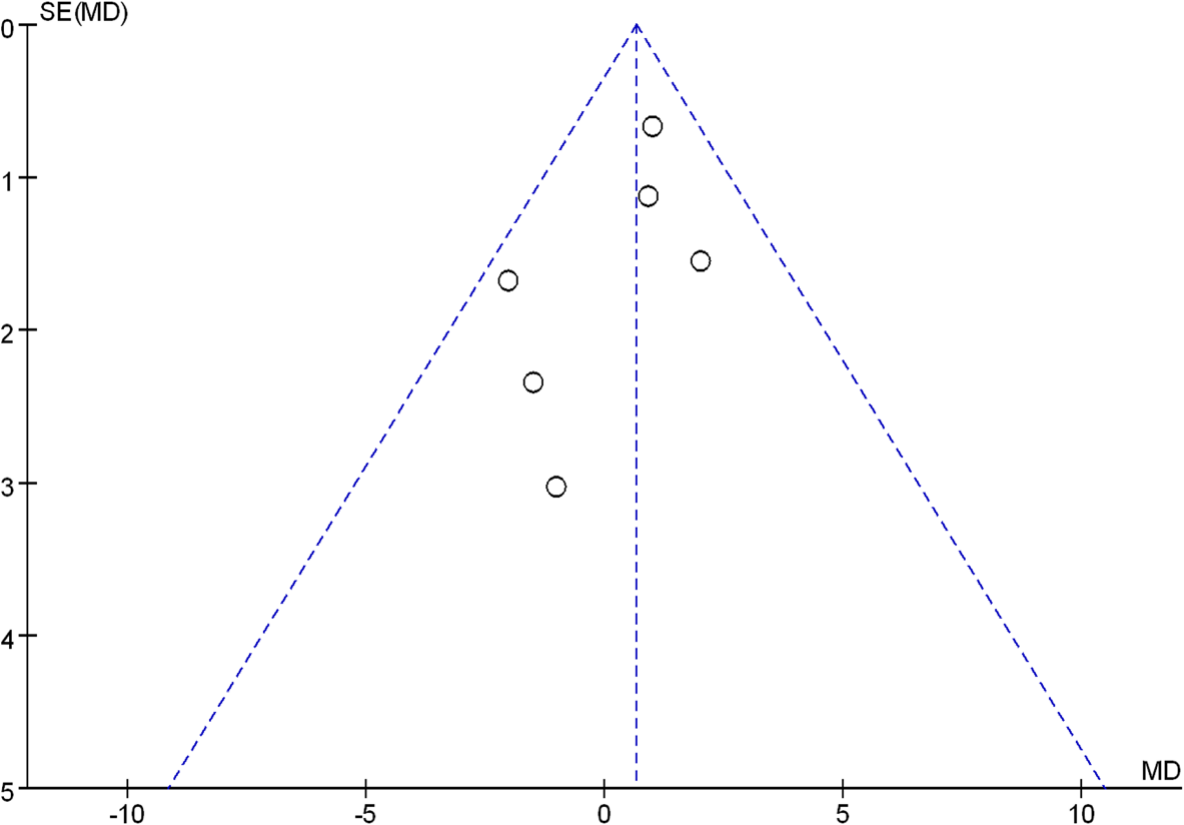
Publication bias

The funnel plot of KSS score was symmetrical, which indicated a low risk of publication bias (Fig. 9). However, publication bias cannot be excluded because positive results were more likely to be published. Quality evidence of the meta-analysis was assessed by the GRADE system. The overall evidence was high, which indicated that further research is unlikely to alter confidence in the effect estimate (Table 3).

**Table 3:** The GRADE evidence quality

Quality assessment						No. of patients		Effect	Quality	Importance
No. of studies	Design	Limitations	Inconsistency	Indirectness	Imprecision	Cementless groups	Cemented groups			
Functional recovery										
6	RCT	No serious limitations	No serious inconsistency	No serious indirectness	No serious limitations	229	255	WMD − 0.239; 95% CI − 2.154 to − 1.676	High	Critical
Range of motion										
4	RCT	No serious limitations	No serious inconsistency	No serious indirectness	No serious limitations	201	199	WMD − 5.284; 95% CI − 9.430 to − 1.139	High	Critical
Pain score										
4	RCT	No serious limitations	No serious inconsistency	No serious indirectness	No serious limitations	140	141	WMD − 3.029; 95% CI − 5.119 to − 0.939	High	Critical
Radiological outcomes										
6	RCT	No serious limitations	No serious inconsistency	No serious indirectness	No serious limitations	229	255	RD 0.058; 95% CI 0.004 to 0.111	High	Critical
Aseptic loosening										
5	RCT	No serious limitations	No serious inconsistency	No serious indirectness	No serious limitations	194	221	RD − 0.001; 95% CI − 0.030 to 0.028	High	Critical
Postoperative complications										
6	RCT	No serious limitations	No serious inconsistency	No serious indirectness	No serious limitations	229	255	RD 0.007; 95% CI − 0.019 to 0.033	High	Critical

## Discussion

To the best our knowledge, this is the first meta-analysis from RCTs to compare the clinical outcomes between cementless and cemented fixation after TKA. The most important result was that cementless TKA was associated with better clinical outcomes such as pain score and radiological outcomes compared to cemented TKA in young patients. No significant difference was found regarding the KSS, range of motion, aseptic loosening, or complications. The overall evidence was high, which indicated that further research is unlikely to alter confidence in the effect estimate.

With the aging population, osteoarthritis (OA) is more and more common. It was reported that more than 50 million patients suffered from knee OA in the USA and the annual workload of TKA procedures was expected to reach 3.5 million by 2030 [16]. It has been a public health issue. TKA was widely accepted to be performed for patients aged 60 years or older. However, it was controversial in young patients. It is known that young patients with TKA have a higher demand of mechanical strength and stability, and the optimal type of prosthesis remains controversial for young patients. Duffy et al. [17] showed that implant survival rate was estimated to be 96% at 10 years and 85% at 15 years of follow-up for patients aged less than 60 years. Functional outcome is an important parameter after TKA, and it is extremely crucial for young patients. Mont et al. [18] reported no clinical differences between the cementless and cemented replacements in young patients at a mean follow-up of 7 years, but it was a retrospective trial. In our study, KSS function and range of motion were used to assess the function recovery after TKA. The present meta-analysis indicated that there was no significant difference between groups after TKA.

Standard anteroposterior and lateral X-ray plain film with the beam tangential to the tibial component were used to describe the presence and size of radiolucent lines at the tibial component interface as introduced by the Knee Society and to assess potential osteolytic lesions. Previous studies indicated that radiolucent lines may be associated with loosening or instability, including migration, and inadequate load distribution. Aebli et al. [19] reported that radiolucent lines may occur because of the imperfect cuts of the tibial plateau or micromotions leading to the formation of gaps, which may prevent osteointegration in cementless TKA. Smith et al. [20] showed that the radiolucency lines around the tibial component were due to a failure to inject cement into the sclerotic bone. Huddleston et al. [21] found that when excellent initial stability was obtained, uncemented femoral fixation yielded fewer radiolucent lines in the posterior femoral condylar region compared with cemented fixation. Rand [22] reported that radiolucent lines adjacent to the tibial component were similar in both groups. In our study, all RCTs with 484 patients reported the radiological outcomes. The present meta-analysis indicated that cementless fixation was associated with a significantly improved radiological outcome compared with the cemented group.

Pain control after TKA is important and has become a serious clinical problem. It was reported that about 50% of TKA patients suffered moderate to severe postoperative pain. Effective analgesic method enhances the knee functional recovery and reduces hospitalization days. Opioid is commonly used for pain relief; however, several side effects including nausea, vomiting, constipation, and urine retention may affect the prognosis [23, 24]. To reduce opioid consumption, several analgesic strategies have been used. Mutsuzaki et al. [25] showed that there was a close relationship between tibial radiolucent lines and continual moderate knee pain. The present meta-analysis indicated that cementless fixation was associated with a significantly improved pain score after TKA. The mechanism is still unclear; we hypothesized that cementless prosthesis achieved stable fixation, while the cemented prosthesis may have progressive defects of fixation, and this may result in pain and mobility, although no evidence of loosening was found.

Aseptic tibial loosening is a common complication. Nilsson et al. [10] indicated that the incidence of loosening is similar between cementless and cemented fixation. Previous studies have reported that rates of aseptic tibial loosening is 0 to 1% in cementless implants compared to 1 to 12% in cemented components [26, 27]. However, most studies have analyzed the elderly patients with TKA. Young patients required higher demand of daily activities, and perhaps there were higher rates of loosening. Thus, more mechanical complications and potential revisions could be expected over time. In our study, 5 RCTs reported the rate of loosening after TKA. The present meta-analysis indicated that there was no significant difference between cementless and cemented fixation regarding the aseptic tibial loosening. Deep infection is a catastrophic complication following joint replacement which may cause reoperation and huge medical cost. Deep infection after TKA was diagnosed in 4 of the 229 knees (2%) in the cementless group and 2 of 255 knees (1%) in the cemented group. The rates of deep infection and reoperation were comparable regardless of the fixation. Thrombotic complication is severe and may develop to pulmonary embolism. No significant difference was found between the groups.

Although the inclusion criteria for this study were more stringent, there were still some limitations: (1) only six RCTs with small sample size were included, and statistical tests might be insufficient; (2) some studies did not show negative results, and some indicators might have higher heterogeneity; (3) publication bias is unavoidable because the identified language was restricted to English; and (4) combining clinical outcomes from different follow-up time points will introduce heterogeneities and potential biases.

## Conclusion

Cementless TKA was associated with superior outcomes in terms of radiological outcomes and pain score compared with cemented fixation. We found no significant difference regarding the functional outcome or aseptic loosening between groups. High-quality RCTs are still required for further investigation.

## Data Availability

Please contact author for data requests

## Abbreviation

KSS: Knee Society Score
OA: Osteoarthritis
RA: Rheumatoid arthritis
RCT: Randomized controlled trials
ROM: Range of motion
TKA: Total knee arthroplasty
